# Effects of Thioglycolic Acid on *in vivo* Oocytes Maturation in Mice

**DOI:** 10.1371/journal.pone.0023996

**Published:** 2011-09-01

**Authors:** Lei Xia, Shaoying Hou, Xiaomei Ren, Zhuoran Wang

**Affiliations:** 1 Department of Reproductive Medicine, The First Affiliated Hospital of Harbin Medical University, Harbin, China; 2 Department of Nutrition and Food Hygiene, School of Public Health, Harbin Medical University, Harbin, China; Brunel University, United Kingdom

## Abstract

**Background:**

Thioglycolic acid (TGA) is widely used in the hairdressing industry, which mostly caters to women. Recently, TGA has been reported to impair several organs, especially reproductive ones such as testes and ovaries. The reproductive toxicity of TGA on females has become an issue that cannot be neglected.

**Methodology/Principal Findings:**

In the present work, superovulated female mice were percutaneously treated with different doses of TGA (37.81, 75.62, and 151.25 mg/kg). The mice were sacrificed to collect ovulated oocytes, whose numbers were counted and compared. Immunofluorescence-stained oocytes were observed under a confocal microscope to investigate the effects of TGA on spindle morphology, distribution of cortical granules (CGs), and parthenogenetic activation. The number of ovulated oocytes was decreased by TGA. The ovulated oocytes in the 151.25 mg/kg TGA group were significantly less than in the control and in the 37.81 mg/kg TGA groups. The ovulated oocytes in the 75.62 mg/kg TGA group were less than in the 37.81 mg/kg dose group. Abnormal spindle configuration *in vivo* was also induced by TGA. The spindle areas in the 75.62 and 151.25 mg/kg TGA groups were significantly larger than in the control and 37.81 mg/kg TGA groups. The parthenogenetic activation of ovulated oocytes *in vitro* was inhibited as well. The percentage of activated oocytes in the 75.62 and 151.25 mg/kg TGA groups was significantly lower than in the control and 37.81 mg/kg TGA groups. The percentage in the 151.25 mg/kg TGA group was also less than in the 75.62 mg/kg group. CG distribution was not affected by TGA.

**Conclusion:**

Mice were percutaneously treated with TGA. Consequently, the number of ovulated oocytes decreased, abnormal spindle configurations were induced, and the parthenogenetic activation of ovulated oocytes was inhibited. CG distribution was not affected.

## Introduction

Hairdressers are exposed to a variety of chemicals everyday [1]. Chemical waving, a very common hairstyling procedure, uses permanent-waving solutions (PWSs). The active ingredient of PWSs is thioglycolic acid (TGA), which is highly toxic. Hence, concerns on the use of PWSs have arisen given the escalating popularity of hairdressing over the last few decades. Hairdressers are frequently in contact with high amounts of TGA through the skin, inhalation, and even ingestion. Results of animal studies have indicated that contact with PWSs could lead to acute toxicity, such as irritation and burning of the eye, nose, throat, and lungs. Chronic toxicity, which lasts for months, could also occur. Frequent contact with PWSs has been reported to induce allergic reactions, immunological dysfunctions, and mutageneses. The risk of having a small-for-gestational-age infant [2]–[4] in hairdressers also increases. Indeed, intensive exposure to TGA potentially damages the health of both hairdressers and their customers. Percutaneous TGA administration in mice has been found to cause damages to the liver and DNA, signifying the possible mutagenic property of TGA [1], [5]. Other reports indicated that TGA exposure alters the ultrastructures of ovaries and testes, decreases the number of sperm, disturbs the estrous cycle in rats [6], and increases menoxenia in hairdressers [7]. TGA was found to be harmful to animal offspring as well [8]. In our previous studies, TGA was found to inhibit the *in vitro* maturation of mice and *Xenopus* oocytes [9], [10]. However, information on female *in vivo* reproductive toxicity of TGA is not yet available. The purpose of the present study is to investigate the effect of percutaneous TGA treatments on mouse oocyte maturation *in vivo*, and to observe the related phenomena.

## Materials and Methods

### Chemicals

Pregnant mare serum gonadotropin (PMSG) and human chorionic gonadotropin (hCG) were from the Ningbo Second Hormone Factory (China). Bovine serum albumin (BSA) was obtained from Promega (USA). Triton X-100 was purchased from Ameresco (USA). Rabbit anti-mouse tubulin antibody was purchased from the Lab Vision Corporation (USA), and fluorescein isothiocyanate (FITC)-conjugated goat anti-rabbit IgG was from the Zhongshan Goldenbridge Biotechnology Co. Ltd. (China). CZB medium was formulated as previously described [11]. Unless noted, all other chemicals were from Sigma.

### Animals

Female Kunming mice were provided by the Experimental Animal Center of the Second Hospital of the Harbin Medical University. The mice were maintained under the conditions of a 14L∶10D photoperiod, constant temperature, and 65% relative humidity. Food and water were available *ad libitum*. All animal care and experimental procedures were performed in compliance with the policies on the care and use of animals of the Ethical Committee of the Harbin Medical University. The mice were allowed to adapt for at least 1 week in the experimental conditions, and were then randomly assigned into four groups. A 2 cm×2 cm area on the back each mouse was shaved for TGA treatment. The mice were superovulated with an intraperitoneal injection of 10 IU PMSG followed by 10 IU hCG after 48 h.

### TGA treatment

Based on earlier studies, the median lethal dose (LD_50_) for percutaneous TGA administration into mice was 1210 mg TGA/kg body weight [12]. Different body weight doses were designed for the experiments to investigate whether relatively low TGA doses, which theoretically cause no toxicity, are actually toxic to female reproductive functions. The doses were 37.81 mg/kg (1/32 LD_50_), 75.62 mg/kg (1/16 LD_50_), and 151.25 mg/kg (1/8 LD_50_). TGA solutions of different concentrations were prepared in distilled water not more than 30 min before administration. The solutions were gently smeared (0.05 mL/10 g body weight) percutaneously on the depilated back of each mouse simultaneously with the hCG treatment. After 1 h, the backs of the mice were thoroughly washed with warm water, wiped dry, and coated with Vaseline. The animals were observed all the time throughout the treatment to prevent them from licking and rubbing each other. Using the same method above, the animals in the control group were treated with distilled water. TGA was administered to three mice per group.

### Collection of oocytes

About 13–14 h after hCG injection, the mice were sacrificed via cervical dislocation, and the ovulated oocytes were collected. Cumulus cells were released from the oviductal ampullae by a brief exposure to 0.1% hyaluronidase in M2 medium [13]. The cumulus-free oocytes were then washed in M2 medium three times. Only oocytes with first polar bodies were used for the observation of spindle morphology, CG distribution, and parthenogenetic activation.

### Immunofluorescence staining

Oocyte fixation and labeling were performed according to the method of Zhu *et al.* [14]. Briefly, oocytes were fixed with 4% paraformaldehyde in phosphate-buffered saline (PBS) for 30 min at room temperature. They were then permeabilized in incubation buffer (0.5% Triton X-100 in 20 mM Hepes, pH 7.4, 3 mM MgCl_2_, 50 mM NaCl, 300 mM sucrose) for 15 min at 37°C in an incubator. Blocking in 1% BSA for 1 h and overnight incubation at 4°C with rabbit anti-α-tubulin antibody for spindle staining followed. After three washes in PBS with 0.1% Tween-20 and 0.1% Triton X-100 for 5 min, the oocytes were incubated with FITC-conjugated goat anti-rabbit IgG at 37°C for 1 h. The oocytes of the control group were only incubated with the FITC-conjugated secondary antibody. The total area (in µm^2^) of each spindle was calculated based on the method of Sanfins [15] and on confocal microscope observations:
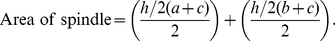

*a*, *b*, and *c* are the widths of different parts of the spindle, and *h* is the total spindle length.

For observing CG distribution, oocytes were first incubated at 37°C in acidified M2 medium for 1–2 min to remove the zona pellucida. After thorough washing in PBS with 0.1% Tween-20 and 0.1% Triton X-100, oocytes were incubated in 100 µg/mL FITC-Lens culinaris agglutinin for 1 h. Following three washes, the cells were incubated in 5 µg/ml propidium iodide (PI) for chromosome staining. After extensive rewashing, the samples were placed in droplets of 1,4-diazobicyclo(2,2,2)octane to avoid photobleaching, and were mounted under coverslips. The mounted samples were kept frozen and protected from light until observation. The oocytes were classified according to CG distribution. Oocytes with CGs arranged in clusters throughout the entire cytoplasms were classified as “immature,” those with CGs only in the periphery were “mature,” and those without CG (CG exocytosis) were “activated.”

### Oocyte activation

**Figure 1:**
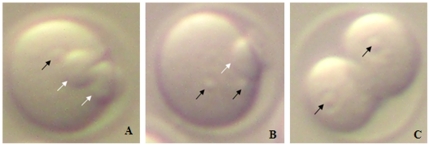
Parthenogenetically activated oocytes (×300) with pronucleus (PN) and polar body (PB). The black arrow shows PN and the white arrow shows PB. A: The activated oocyte with 1 PN and 2 PB. B: The activated oocyte with 2 PN and 1 PB. C: The activated oocyte with 2 cells.

The activating medium was Ca^2+^-free CZB [11] supplemented with 10 mM SrCl_2_. The oocytes were treated with SrCl_2_ for 6 h, stained with 5 µg/ml PI in PBS for 5 min, and microscopically examined for evidence of activation. Oocytes were considered activated when each cell contained one or two well-developed pronuclei, or when two cells have pronuclei (Figure 1).

### Data analysis

At least three replicates were conducted for each treatment. Data were expressed as means ± SD and were analyzed using ANOVA. *P* values less than 0.05 were considered significant.

## Results

### Effect of TGA on the number of ovulated oocytes

Table 1 shows that the average numbers of ovulated oocytes were 32±4, 28±5, and 13±5 after TGA treatment at doses of 37.81, 75.62, and 151.25 mg/kg, respectively. There was no significant difference between the control (38±6) and 37.81 mg/kg TGA (32±4) groups. In contrast, the ovulated oocytes in the 151.25 mg/kg (13±5) TGA group were significantly less than in the control and 37.81 mg/kg TGA groups. The ovulated oocytes in the 75.62 mg/kg TGA group were less than in the 37.81 mg/kg TGA group significantly, but it is not different from the control group.

**Table 1:** Effect of TGA on the number of ovulated oocytes.

Group	Total number of ovulated oocytes (number of experiments)	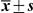
Control	269 (7)	38±6
37.81 mg/kg TGA	260 (7)	32±4
75.62 mg/kg TGA	199 (7)	28±5Δ
151.25 mg/kg TGA	93 (7)	13±5* ^,^ Δ

*Compared to control group; *P*<0.05.

Δ Compared to 37.81 mg/kg TGA group; *P*<0.05.

### Effect of TGA on the spindle morphology of ovulated oocytes

**Figure 2:**
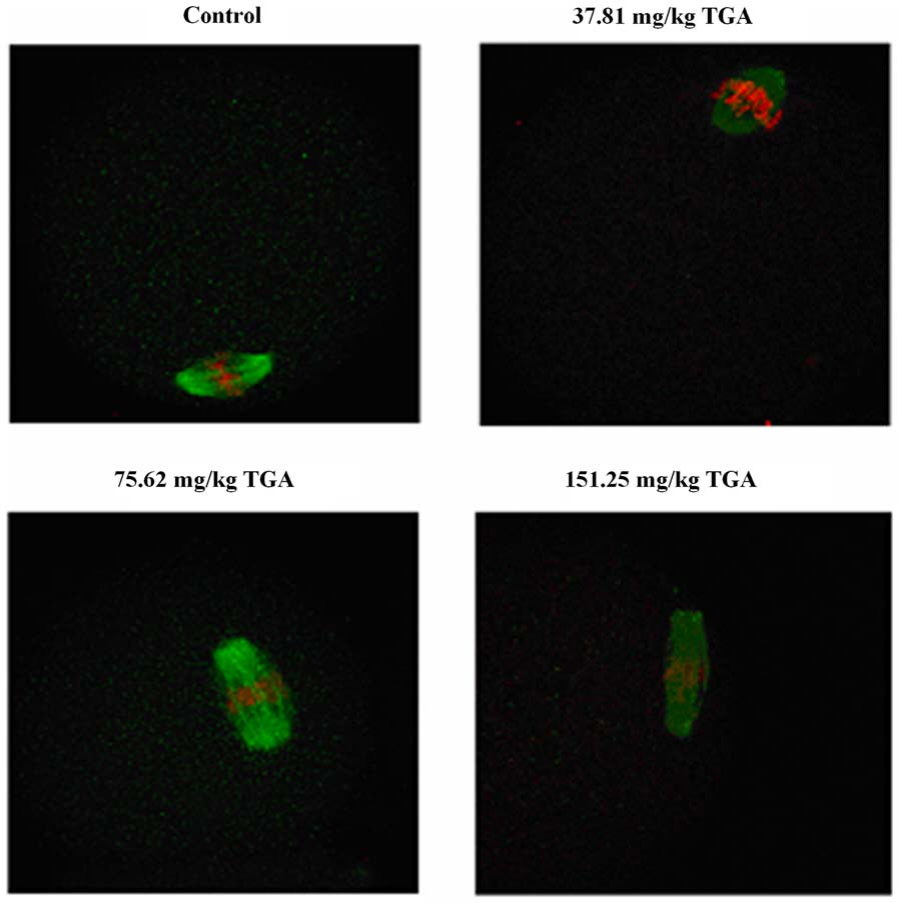
Effect of TGA on the spindle morphology of *in vivo* matured oocytes (×600). The oocytes were stained immunocytochemically with anti-α-tubulin monoclonal antibody and fluoroscein isothiocyanate to observe the spindle (green). Counterstaining with PI was also performed to detect the chromosomes (red).

**Figure 3:**
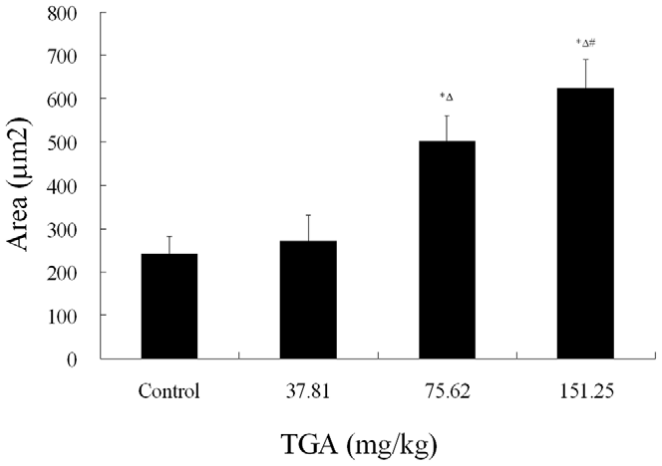
Effect of TGA administration on the spindle area of *in vivo* matured oocytes (*n* = 22). * Compare to control group, *P*<0.05; ^Δ^ Compared to 37.81 mg/kg TGA group, *P*<0.05; ^#^ Compared to 75.62 mg/kg TGA group, *P*<0.05.

Figure 2 shows that the ovulated oocytes displayed bipolar spindles with focused poles in the control and 37.81 mg/kg TGA groups. In contrast, the oocytes displayed a large barrel configuration in the 75.62 and 151.25 mg/kg TGA groups. The spindle areas in the four groups were 241.08±47.48, 271.88±59.66, 502.95±57.03, and 623.02±67.53 µm^2^, respectively (Figure 3). Spindle area significantly increased with increased TGA dose, demonstrating a dose-dependent relationship. The spindle areas in the 75.62 and 151.25 mg/kg TGA groups were both significantly larger than in the control and 37.81 mg/kg TGA groups. The spindle area in the 151.25 mg/kg TGA group was also significantly larger than in the 75.62 mg/kg TGA group.

### Effect of TGA on CG distribution within ovulated oocytes

**Figure 4:**
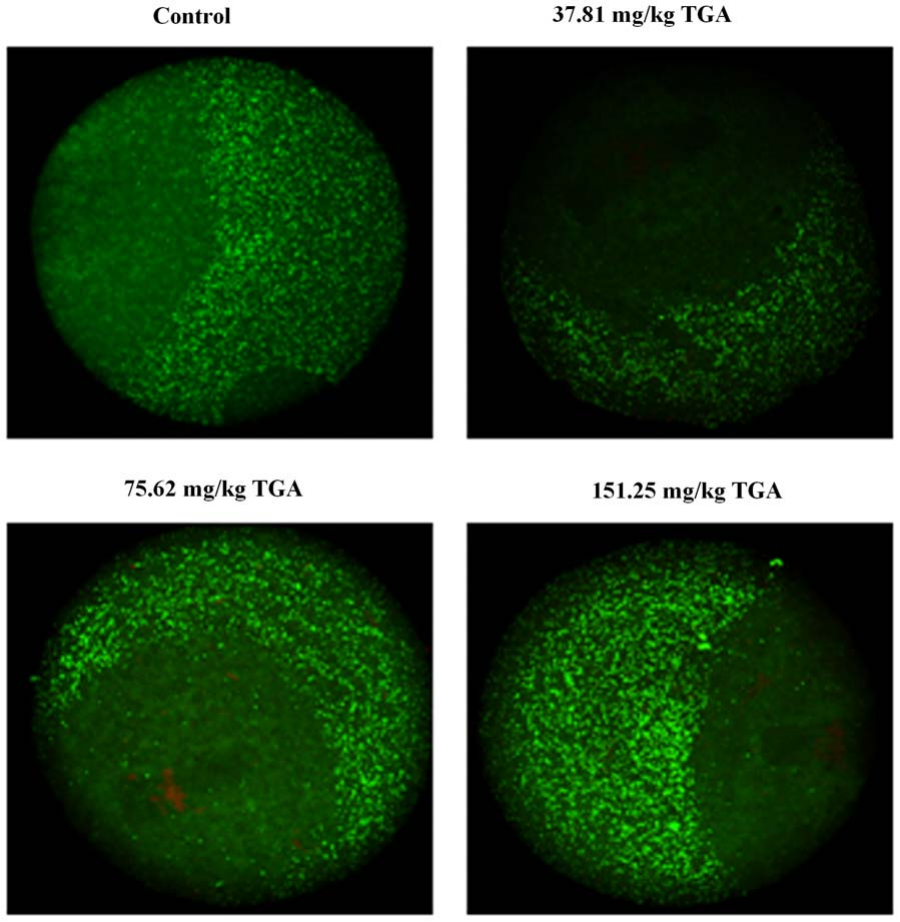
Effect of TGA on the distribution of cortical granules (CGs) of *in vivo* matured oocytes (laser scanning confocal microscopic images, ×600). The oocytes were stained with fluorescein isothiocyanate-Lens culinaris agglutinin. The green fluorescence shows the CG distributions.

CG distribution within the oocytes was observed under confocal microscopy by staining the CGs with immunocytochemicals. Figure 4 shows that after percutaneous TGA treatment in all groups, the CGs migrated to the cortex and formed a continuous layer under the oolemma. A CG-free domain (CGFD) was found, and there was no significant difference among the control and TGA-treated groups.

### Effect of TGA on *in vitro* ovulated oocyte activation

After the oocytes were parthenogenetically activated by 10 mM SrCl_2_, the percentage of activated oocytes decreased with increased TGA dose. Table 2 shows that although treatment with 37.81 mg/kg TGA decreased oocyte activation, this change had no statistical significance. In the 75.62 and 151.25 mg/kg TGA groups, the percentages of activated oocytes were significantly lower than in the control and 37.81 mg/kg TGA groups. The percentage of activation in the 151.25 mg/kg TGA group was also significantly lower than in the 75.62 mg/kg group.

**Table 2:** Effect of TGA on the parthenogenetic activation of oocytes.

Group	Total number of ovulated oocytes (number of experiments)	Activated oocytes(%)
Control	147 (6)	96±0
37.81 mg/kg TGA	144 (6)	90±1.2
75.62 mg/kg TGA	94 (6)	86±3.1* ^,^ Δ
151.25 mg/kg TGA	69 (6)	67±4.9* ^,^ Δ ^,^ #

*Compared to control group, *P*<0.05.

Δ Compared to the 37.81 mg/kg TGA group, *P*<0.05.

# Compared to the 75.62 mg/kg TGA group, *P*<0.05.

## Discussion

Exposure to TGA has become a very common and severely hazardous phenomenon given the rising popularity of hairdressing. Numerous studies have shown that TGA exposure could cause acute and subchronic damage to rats and mice, as well as reproductive toxicity [1], [12]. The latter requires further attention. Our previous study revealed that mice oocytes treated with TGA presented abnormal chromosomal arrangements and spindle configurations, as well as inhibited *in vitro* maturation [9]. TGA inhibited the *in vitro* maturation of *Xenopus* oocytes by increasing germinal vesicle breakdown frequency as well as altering protein expression and phosphorylation involved in the maturation promoting factor (MPF) and mitogen-activated protein kinase (MAPK) pathways [10]. To identify further the effect of TGA on reproductive functions as well as determine possible differences between *in vitro* and *in vivo* experiments, we investigated the activities of TGA *in vivo*.

TGA doses that corresponded to 1/32, 1/16, and 1/8 LD_50_ were percutaneously administered to female mice. The purpose was to investigate whether such low doses related to the experimental LD_50_ could interfere in the *in vivo* development and maturation of mouse oocytes. Our results demonstrated that TGA doses of 1/8 LD_50_ (151.25 mg/kg) and 1/16 LD_50_ (75.62 mg/kg) decreased both the number of ovulated oocytes and the percentage of parthenogenetically activated oocytes, as well as caused abnormal and larger spindles. Actually, the doses we used also were low doses relevant to real-life exposure levels. Chinese national standard for permissible concentration of TGA in permanent-waving solutions (PWSs ) (GB 7916-87) is 8%–10%. According to our treatment method (smearing percutaneously), the highest dose of TGA (151.25 mg/kg) was only close to 3.03%, which is much lower than the practical touching concentration for hairdressers and their customers. Of course, besides TGA concentration, the volume and the duration of using PWSs also influence the real-life exposure levels for human beings.

Several factors are involved in the regulation of ovulation rate, such as intraovarian factors, exogenous hormone administration, and environmental factors. Among these, the hypothalamic-pituitary-ovary axis is believed to be the most important [16], [17]. In the present study, all ovulated oocytes were obtained from superovulated mice, but only after TGA treatment was mice ovulation inhibited. Therefore, we speculate that this inhibition could be attributed to TGA disturbing the hypothalamic-pituitary-ovary axis. However, if such a disturbance did occur, TGA did not completely block gonadotropin-induced superovulation at the doses we used. Moreover, nutrition affects follicle development [18]. Zak et al demonstrated that the size of follicles and the rate of maturation of oocytes obtained from restricted-fed sows could be affected by the nutritonal history [19]. TGA was reported to increase the glucose-6-phosphatase activity and cause protein and carbohydrate metabolism disorders in rat liver [5]. Hence, we propose that these metabolism problems may contribute to the inhibiting effect of TGA on ovulation. Other factors such as ovarian autocrine, paracrine, and endocrine systems may also affect ovulation [20].

Spindle formation and CG distribution are both indicators of oocyte maturation. A meiotic spindle is composed of microtubules and appears from the late G2 phase to the end of the M phase. Meiotic spindles are crucial for normal chromosome alignment as well as for the separation of maternal chromosomes during meiosis I and II [21]. The normal MII spindle has a characteristic barrel shape. Spindles are sensitive to temperature [22], xenobiotic chemicals, and drugs [23]. Recently, researchers used spindle analysis to assess oocyte quality as well as the effects of toxins and drugs on oocytes [24]. The present study showed that *in vivo* TGA treatment induced abnormal, larger-barrel spindles, which agrees with the results of a previous *in vitro* experiment [9]. Although the *in vivo* mechanism of spindle damage is not yet clear, MAPK and MPF may play essential roles in the regulation of microtubule dynamics [25], [26]. The effects of MAPK and MPF signals on *in vitro* oocyte maturation after TGA treatment was observed [27]. This finding makes it rational to speculate that TGA altered the spindle morphology of mouse oocytes through the MAPK and MPF signaling pathways, but the mechanism warrants further investigation.

CGs, which are about 0.2–0.6 µm in diameter, are round and membrane-bound organelles found in many invertebrate and vertebrate oocytes. Oocyte CGs originate from the Golgi apparatus and first appear at the onset of follicular growth. The CGs then migrate to the cortex and form a continuous layer under the oolemma during oocyte growth and maturation [28], [29]. When the oocytes are mature enough, a CG-free domain (CGFD) forms where the plasma membrane lacks microvilli [30]. Previous studies in mice have demonstrated that sperm are less likely to penetrate an egg in the amicrovillar region [31]. Therefore, CGs play a significant role in the blocking of polyspermic penetration in mammalian oocytes [26]. An increasing number of studies have also suggested that CG distribution measures cytoplasmic maturation [32]. In the present study, we detected CG distribution to determine if TGA also affected *in vivo* oocyte cytoplasmic maturation in mice and found that a CGFD still appeared after percutaneous TGA administration. This result was contrary to that observed in *in vitro* matured oocytes, where CG distribution was inhibited and the CGFD disappeared after TGA treatment [27]. This difference may be explained on one hand by the fact that different doses were used in the *in vivo* and *in vitro* experiments, and that a relatively low TGA dose of 1/8 LD_50_ was used in the present study. On the other hand, the metabolism and detoxification of TGA *in vivo* cannot be neglected.

Spindle disturbance may severely affect oocyte development after activation. Therefore, the abnormal spindle configuration that resulted from the TGA treatment in mice suggested that oocyte activation might be compromised to investigate further the following oocoyte fate. When oocytes are activated, there is a series of Ca^2+^ oscillations [33], [34]. In the present paper, a strontium chloride-containing medium was used to induce intracellular Ca^2+^ release that triggers a series of Ca^2+^-dependent biological responses, and results in parthenogenetic activation. Considering that the parthenogenetic activation rate decreased in TGA-treated mice, we propose that TGA may alter Ca^2+^ concentration in oocytes or affect intracellular Ca^2+^ release, although the mechanism remains unclear. Furthermore, the inhibitory effect on the parthenogenetic activation of ovulated oocytes was significant in TGA groups at 75.62 and 151.25 mg/kg, in consistence with the effect on spindle area. Hence, we speculate that the decreased oocyte activation may have resulted from spindle formation perturbation caused by TGA. We also hypothesize that TGA affects the fertilization process, but further studies are needed.

In conclusion, we found that *in vivo* TGA treatment decreased the number of ovulated oocytes and induced abnormal spindle configurations. TGA also inhibited the parthenogenetic activation of ovulated oocytes. TGA toxicity to reproductive functions deserves further investigations.
